# Contextual factors influencing the equitable implementation of precision medicine in routine cancer care in Belgium

**DOI:** 10.1093/eurpub/ckae055

**Published:** 2024-03-28

**Authors:** Tugce Schmitt, Marie Delnord, Emilie Cauët, Els Van Valckenborgh, Marc Van den Bulcke

**Affiliations:** Cancer Centre, Department of Epidemiology and Public Health, Sciensano, Brussels, Belgium; Cancer Centre, Department of Epidemiology and Public Health, Sciensano, Brussels, Belgium; Cancer Centre, Department of Epidemiology and Public Health, Sciensano, Brussels, Belgium; Cancer Centre, Department of Epidemiology and Public Health, Sciensano, Brussels, Belgium; Cancer Centre, Department of Epidemiology and Public Health, Sciensano, Brussels, Belgium

## Abstract

**Background:**

Precision medicine represents a paradigm shift in health systems, moving from a one-size-fits-all approach to a more individualized form of care, spanning multiple scientific disciplines including drug discovery, genomics, and health communication. This study aims to explore the contextual factors influencing the equitable implementation of precision medicine in Belgium for incorporating precision medicine into routine cancer care within the Belgian health system.

**Methods:**

As part of a foresight study, our approach evaluates critical factors affecting the implementation of precision oncology. The study scrutinizes contextual, i.e. demographic, economic, societal, technological, environmental, and political/policy-related (DESTEP) factors, identified through a comprehensive literature review and validated by a multidisciplinary group at the Belgian Cancer Center, Sciensano. An expert survey further assesses the importance and likelihood of these factors, illuminating potential barriers and facilitators to implementation.

**Results:**

Based on the expert survey, five key elements (rising cancer rates, dedicated healthcare reimbursement budgets, increasing healthcare expenditures, advanced information technology solutions for data transfer, and demand for high-quality data) are expected to influence the equitable implementation of precision medicine in routine cancer care in Belgium in the future.

**Conclusions:**

This work contributes to the knowledge base on precision medicine in Belgium and public health foresight, exploring the implementation challenges and suggesting solutions with an emphasis on the importance of comparative analyses of health systems, evaluation of health technology assessment methods, and the exploration of ethical issues in data privacy and equity.

## Introduction

Precision medicine represents a paradigm shift in health systems, moving from a one-size-fits-all approach to a more individualized form of care, spanning multiple scientific disciplines including drug discovery, genomics, and health communication. Rooted in data-driven decision-making, it seeks to offer treatments tailored to each patient’s unique health status, thereby influencing their healthcare journey.^1^^,^^2^ Essentially, in precision medicine up-to-date patient information guides the action, which could involve selecting a specific drug, determining its dosage, or even suggesting lifestyle changes.^1^ Precision oncology, a specialized branch of precision medicine, encompasses the diagnosis, treatment, and prevention of cancer by tailoring medical care to the individual characteristics of each patient and tumor. The approach combines multi-modal or multi-omics data to make patient-specific treatment decisions, an advancement that holds the potential to revolutionize cancer care. Large datasets generated by these diagnostic approaches have led to the development of novel techniques and tools for effective data processing and decision-making.^3^ In this context, data-driven decision-making tools supported by artificial intelligence (AI) technologies can also offer unique predictive capabilities, holding the promise to enhance the personalization of cancer care.^4^^,^^5^

European health systems strive for widespread implementation of precision medicine, enabling its benefits to be accessible to a broad population.^6^ The European Commission sees precision medicine as an effective means of addressing cancer and implementing tailor-made prevention and treatments that work without wasting resources in trial-and-error treatments.^7^ Indeed, a key focus of the Europe’s Beating Cancer Plan (EBCP) is to prioritize access to precision oncology, a field that tailors cancer treatment and prevention strategies based on individual genetic profiles.^7^ By facilitating access to precision oncology and promoting the collaborative exchange of genomic information, EBCP aims to drive advancements in cancer care and foster equitable health outcomes throughout the European Union (EU) and actively encourages the widespread sharing of genomic data on a large scale.^8^ Among many other initiatives in the pipeline, the Commission supports EU Member States with several actions in the field of precision oncology such as (i) research to identify the genetic predisposition of individuals to develop cancers for personalized risk-assessment and targeted cancer prevention; (ii) ‘Cancer diagnostic and treatment for all’ to improve access to innovative cancer diagnosis and treatment by using the Next-Generation Sequencing (NGS) technology and (iii) the European Cancer Imaging Initiative to make anonymized images accessible to hospitals, researchers, and innovators.^7^ Precision oncology could help lessen the burden of cancer in Europe, caused by aging populations, unhealthy lifestyles, and unfavorable health determinants.^9^

Similar to other European countries, also in Belgium the burden of cancer survivorship has been increasing over the years. In particular, breast, colorectal, and non-melanoma skin cancers are notable in their morbidity impact among the Belgian population.^10^ Precision oncology could provide better outcomes and lessen the strain on healthcare resources by ensuring more targeted and effective cancer therapies, minimizing unnecessary treatments, and thereby enhancing cost-effectiveness in the long term. Yet, challenges occur when aiming for implementing precision oncology at the country level without exacerbating existing inequalities in the health system. As advancements in genomics and precision medicine continue to evolve, the absence of robust public health action combined with an equity-focused approach could risk widening health disparities among the population.^9^^,^^11^ Ensuring equal access to cutting-edge treatments, representative genetic data, and high data security standards is imperative in this context. When planning the implementation, thorough consideration should also be given to the short- and long-term economic implications of precision oncology. Literature suggests that budget allocations for patient reimbursement and funding for cost-effectiveness studies should be integral to the equitable dissemination of precision oncology benefits.^12^ In the absence of overarching and sustainable funding in the health system, the deployment of precision oncology could be unrealistic to implement in less affluent regions, raising questions about its broader applicability and ethical use.

This study aims to explore the main contextual factors that facilitate or hinder the implementation of precision medicine in routine cancer care in the Belgian health system in an equitable way. As introduced above, precision oncology has transformative potential in healthcare; however, its implementation requires time and fundamental changes in health systems.^13^ Hence, although new technologies and precision medicine strategies can improve cancer prevention and treatment, there is much space for translation of research into practice, including in Belgium.^14^ Arguably, when looking to integrate precision oncology into routine cancer care at the country level, a multi-faceted approach is required. Indeed, when developing and implementing new health service policies, the literature suggests that it is essential to consider evidence from scientific research in combination with various contextual factors, spanning from legal aspects to economic influences.^15^ Hence, the implementation approach towards precision oncology should include discussions not only on advancements in technological capabilities but also a re-evaluation and potential redesign of healthcare policies and systems. Above all, equitable implementation at the country level demands comprehensive frameworks for reimbursement and ethical considerations. With this study, we shed light on the emerging topic of precision oncology and its implementation in routine cancer care in Belgium from different contextual perspectives.^16^

## Methods

Foresight, within the domain of public health, is a systematic and participatory process aimed at envisioning the future, anticipating trends, and supporting present-day actions in the field of public health.^17^ Interest in incorporating foresight methodology into public health is on the rise and has been gaining widespread adoption, especially for innovative fields such as precision medicine.^18^ While frameworks exist for the implementation of genomic-medicine programs within individual institutions and multi-institutional collaborations, there is limited information available on how to effectively translate this experience into comprehensive transformations of entire health systems.^19^ Considering the advantages that precision medicine can bring to cancer prevention, diagnosis, and treatment and acknowledging at the same time the trends happening outside of the field of oncology or even health systems, which can act as drivers or barriers in the use of precision oncology in Belgium, this study employed a foresight methodology to reach its aim. Undertaking a foresight study is crucial for several reasons: It equips the health system for future challenges, provides insights into innovations, and offers strategies to manage the escalating costs associated with cancer care. Ultimately, this forward-looking analysis can facilitate evidence-based decision-making and strategic planning in response to the evolving genomics landscape in Belgium.

**Figure 1.**
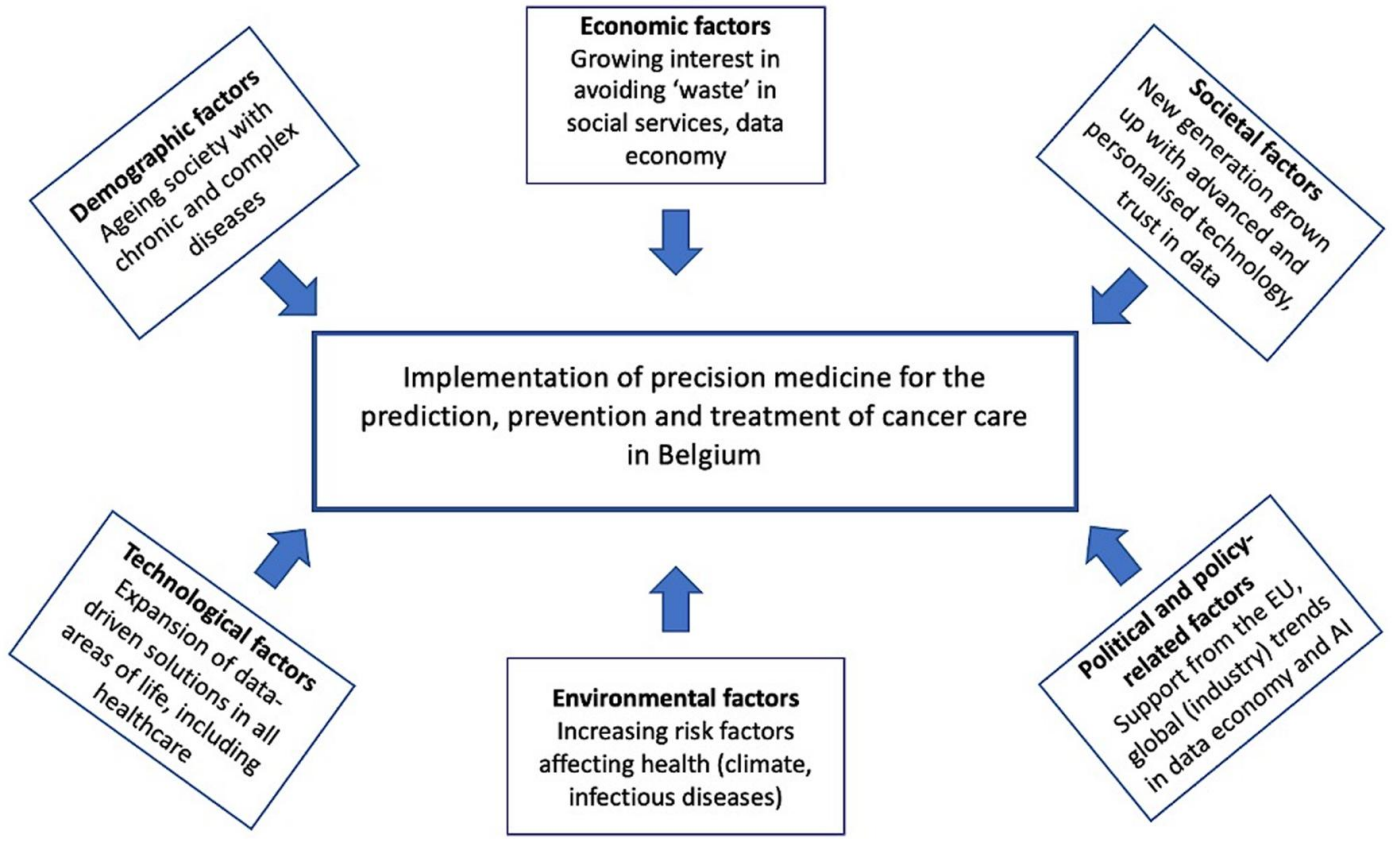
Conceptual framework

Our methodology has been guided by the Work Package ‘Foresight: Modelling and Scenarios’ of the European project Population Health Information Research Infrastructure (PHIRI). The PHIRI project recognizes that comprehension of potential future developments is important for policymakers to anticipate and influence trends, such as the delivery of regular healthcare services, lifestyle changes, and socio-economic developments.^17^ A crucial component of conducting foresight research is to identify the main driving forces and barriers that influence the topic at hand. As suggested by PHIRI, the DESTEP framework, which encompasses trends in demography, economy, society, technology, environment, and politics/policy, is a commonly used approach to identify specific contextual factors. Based on a conceptual framework created (figure 1), factors influencing the implementation of precision oncology in Belgium were assessed, as explained below.

As the first step of this foresight study, we conducted a literature review. In our scoping review in PubMed/MEDLINE, we used the search query (‘personalized medicine’ OR ‘personalized medicine’ OR ‘personalized healthcare’ OR ‘personalized healthcare’ OR ‘personalized health care’ OR ‘personalized health care’ OR ‘precision medicine’ OR genomics) AND (cancer OR oncology) AND ‘Belgium’ AND ‘implementation’ to screen the abstracts of the papers in English and were published in the last five years. This search strategy yielded 69 papers, 13 of which were relevant to the aim of our study. The 13 peer-reviewed publications were snowballed and enriched with gray literature, which yielded 29 more publications. In total, 42 publications were scrutinized to explore the contextual factors affecting the implementation of precision medicine in Belgium according to the DESTEP framework. To ensure comprehensive coverage of relevant influential factors, a multidisciplinary group at the Belgian Cancer Centre, Sciensano, reviewed and validated the outcomes, which resulted in 46 influential factors.

As the next step, the 46 factors relevant to the implementation of precision medicine in Belgium were evaluated by country experts. The country experts belonged to the Belgian EBCP Mirror Group which includes policy institutions, professional groups, patient associations, academia, and industry.^20^ A survey was administered to them to assess the importance and likelihood of the 46 identified factors. As per the foresight methodology, the aim of our survey was two-fold: Ranking the importance (‘How important are these factors for the implementation of precision medicine in routine cancer care in Belgium in a fair and equitable way?’) and the likelihood (‘How high is the chance that these factors will influence the implementation of precision medicine in routine cancer care in Belgium in a fair and equitable way?’) of the contextual factors identified from the literature. The two-fold objective was crucial for a nuanced understanding of the landscape for implementing precision medicine in routine cancer care in Belgium: (i) assessing the importance of each factor provided insights into what stakeholders perceive as critical elements for successful implementation, and (ii) evaluating the likelihood of each factor influencing the implementation served to anticipate potential barriers or facilitators in the landscape.

A total of 38 experts (table 1) participated in the web-based survey that ensured the anonymity of respondents. They ranked the importance and likelihood of the influential factors based on their perceived impact on the implementation of precision medicine in routine cancer care in Belgium. Notably, it cannot be ruled out that the survey participants were not fully representative of all Belgian cancer stakeholders, similar to the EBCP Mirror Group itself. The reason why we nonetheless used the EBCP Mirror Group as a platform was to engage with a pool of precision medicine experts who are familiar with Sciensano’s work on cancer care in Belgium and thus more likely to contribute to our study, rather than to obtain a comprehensive representation of the group. As the final step of the foresight study, the experts elaborated on the survey outcomes in two online workshops, which yielded qualitative outcomes and will be detailed in another study. The next section presents survey results assessing the importance and likelihood of 46 influential factors, using a five-point Likert scale where 1 represents the lowest and 5 is the highest score.

**Table 1:** Experts participating in the survey

Field	Number of experts
Academia/research organization	12
University hospital	9
Industry	4
Hospital	3
Health insurance fund	3
Patient organization	3
National cancer registry	1
National public health institute	1
Foundation	1
Professional society of oncology	1
**Total**	**38**

## Results

The survey results indicate that several contextual factors play a crucial role in the implementation of precision oncology in Belgium in a fair and equitable way (table 2). These factors, in total eight (Q2 equal to or scored higher than 4,5 in table 2) out of 46, encompass mostly the economic and technological considerations: Increasing incidence and prevalence of cancer; allocated budget for reimbursement of personalized healthcare in the health system, especially in the post-pandemic time; allocated budget for running studies on (the cost-effectiveness of) personalized medicine and translational research; rising healthcare expenditure in health systems; information technology (IT) solutions that allow the transfer of relevant data to a uniform, secure technical platform; quality of data; semantic interoperability and data integration and harmonization; standardization (of protocols, tests and nomenclature) across hospitals and laboratories. Looking at the future, three out of these eight key factors are projected to have a slightly lower probability of impacting the implementation (Q2 lower than 4,5 in table 3) compared to the other five factors: Allocated budget for running studies on (the cost-effectiveness of) personalized medicine and translational research; semantic interoperability and data integration and harmonization; standardization (of protocols, tests and nomenclature) across hospitals and laboratories (table 3).

**Table 2:** Survey results for importance; highlight on the most important items

DESTEP factors	Items	Min.	Q1	Q2 (Median)	Mean	Q3	Max.
		(lowest value: 1, highest value: 5)					
Demographic factors	Aging society	1	3	4	3,74	5	5
	Globalizing society	1	2	3	2,84	4	5
	Genetic diversity in the population	1	3	3	3,47	4	5
	Migration, displacement, refugees	1	2	3	2,68	4	4
	Educational level of the population	1	3	3	3,21	4	5
	Increasing incidence and prevalence of cancer	2	4	5	4,37	5	5
Economic factors	Allocated budget for reimbursement of personalized healthcare in the health system, especially in post-pandemic time	3	4	5	4,63	5	5
	Allocated budget for running studies on (the cost-effectiveness of) personalized medicine and translational research	1	4	5	4,18	5	5
	Allocated budget for data-storage, analysis and interpretation after NGS	2	3	4	4,05	5	5
	Allocated budget for training workforce for personalized medicine (healthcare professionals and researchers)	2	3,25	4	4,11	5	5
	Rising healthcare expenditure in health systems	2	4	5	4,32	5	5
	Competitive global NGS market	2	3	4	3,89	4,75	5
	Decreasing costs for NGS in health systems	3	4	4	4,24	5	5
Societal factors	Socio-economic inequalities in the population for receiving quality healthcare services	2	3	3,5	3,61	5	5
	Health literacy: Patient information for treatments that involve new technologies	1	3	4	3,66	5	5
	Citizen engagement through focus group studies and citizen labs	1	2	3	2,87	3	5
	Citizens’ trust to foster the exchange of information and intelligence	1	3	3,5	3,50	4	5
	Ethical standards for clinical research and data-driven research initiatives	2	3	4	3,76	5	5
	Spread of disinformation through websites and social media channels	1	2	3	3,08	4	5
	Multistakeholder engagement incl. health-care professionals, policymakers, payers, advocacy groups, researchers	2	3,25	4	4,16	5	5
	Peer groups and healthcare professionals embedded in communities to collect data and create tailored education to patients and healthcare professionals	1	3	4	3,79	4,75	5
Technological factors	Digitalization and automation trend that fosters health data exchange and supports research on new preventive care strategies and treatments	2	4	4	4,21	5	5
	IT solutions that allow the transfer of relevant data to a uniform, secure technical platform	2	4	5	4,26	5	5
	Endorsement of clinical decision support tools	2	3	4	4,03	5	5
	Issues concerning the reliability of artificial intelligence (AI) systems	1	3	4	3,87	5	5
	Quality of data	3	5	5	4,74	5	5
	Technical infrastructure for population-wide risk assessment and data collection	2	3	4	3,92	5	5
	Semantic interoperability and data integration and harmonization	2	4	4,5	4,21	5	5
	Supportive technical and logistical environment for clinical trials in precision oncology	2	3	4	3,95	5	5
	Standardization (of protocols, tests, and nomenclature) across hospitals and laboratories	2	4	5	4,34	5	5
	Technical and logistical requirements for Next-Generation Sequencing (NGS)	2	3,25	4	4,13	5	5
	Companion diagnostics	1	3,25	4	4,11	5	5
	Federated data infrastructures	1	3	4	3,89	5	5
Environmental factors	Climate change	1	1	2	1,97	3	4
	Pollution (air, water) and housing	1	1	2	2,34	3	5
	Occupational hazards	1	1	2	2,24	3	5
	Geographical location of citizens (infrastructural differences between regions)	1	3	3	3,24	4	5
	Concerns about the negative environmental impact of digital solutions	1	2	2,5	2,58	3	5
Political and policy-related factors	Democracy, the rule of law and fundamental rights that shape dignity, freedoms, equality and solidarity, and the citizens’ rights and justice	2	3	4	3,79	5	5
	Synergies from EU Policies against cancer (e.g. Europe’s Beating Cancer Plan or EU Council recommendations) and similar projects around the world	3	4	4	4,21	5	5
	National and international collaborations and partnerships	3	4	4	4,16	5	5
	Data protection laws (e.g. legal issues related to achieving broad patient consent and data sharing)	2	3,25	4	4,05	5	5
	National or EU policies that foster a secure environment for (collection, curation, analysis, storing, etc.sharing genomics data with relevant organizations, including cross-border	2	4	4	4,08	5	5
	Policies to protect the public from stigmatization and discrimination	1	3	3	3,45	4	5
	Translational research agenda in countries, e.g. through Comprehensive Cancer Centres in countries, and implementation of clinical guidelines	2	4	4	4,08	5	5
	Accountability and liability issues concerning the clinical decision support tools using AI	2	3	4	3,76	4	5

**Table 3:** Survey results for likelihood; highlight on the likelihood of the eight most important items

DESTEP factors	Items	Min.	Q1	Q2 (Median)	Mean	Q3	Max.
		(lowest value: 1, highest value: 5)					
Demographic factors	Aging society	1	4	4	3,92	5	5
	Globalizing society	1	2	3	3,08	4	5
	Genetic diversity in the population	1	3	3	3,32	4	5
	Migration, displacement, refugees	1	2	3	2,74	3,75	5
	Educational level of the population	1	3	4	3,37	4	5
	Increasing incidence and prevalence of cancer	2	4	5	4,37	5	5
Economic factors	Allocated budget for reimbursement of personalized healthcare in the health system, especially in post-pandemic time	3	5	5	4,74	5	5
	Allocated budget for running studies on (the cost-effectiveness of) personalized medicine and translational research	1	3	4	4,03	5	5
	Allocated budget for data-storage, analysis and interpretation after Next-Generation Sequencing (NGS)	2	3	4	3,92	5	5
	Allocated budget for training workforce for personalized medicine (healthcare professionals and researchers)	2	3	4	3,89	5	5
	Rising healthcare expenditure in health systems	3	4	5	4,55	5	5
	Competitive global Next-Generation Sequencing (NGS) market	1	3	4	3,71	4,75	5
	Decreasing costs for Next-Generation Sequencing (NGS) in health systems	2	4	4	4,16	5	5
Societal factors	Socio-economic inequalities in the population for receiving quality healthcare services	1	3	3	3,39	4	5
	Health literacy: Patient information for treatments that involve new technologies	1	2	3	3,24	4	5
	Citizen engagement through focus group studies and citizen labs	1	2	3	2,74	3	5
	Citizens’ trust to foster the exchange of information and intelligence	1	3	3	3,18	4	5
	Ethical standards for clinical research and data-driven research initiatives	1	3	3	3,50	4	5
	Spread of disinformation through websites and social media channels	1	2	3	2,92	4	5
	Multistakeholder engagement incl. health-care professionals, policymakers, payers, advocacy groups, researchers	2	3	4	3,95	5	5
	Peer groups and healthcare professionals embedded in communities to collect data and create tailored education to patients and healthcare professionals	1	3	4	3,74	4	5
Technological factors	Digitalization and automation trend that fosters health data exchange and supports research on new preventive care strategies and treatments	2	3	4	4,05	5	5
	IT solutions that allow the transfer of relevant data to a uniform, secure technical platform	2	3	4,5	4,18	5	5
	Endorsement of clinical decision support tools	2	3	4	3,76	5	5
	Issues concerning the reliability of AI systems	2	3	4	3,74	4,75	5
	Quality of data	2	3,25	5	4,26	5	5
	Technical infrastructure for population-wide risk assessment and data collection	2	3	4	3,82	5	5
	Semantic interoperability and data integration and harmonization	2	3	3,5	3,68	5	5
	Supportive technical and logistical environment for clinical trials in precision oncology	1	3	4	3,63	4	5
	Standardization (of protocols, tests, and nomenclature) across hospitals and laboratories	2	3	4	4,00	5	5
	Technical and logistical requirements for Next-Generation Sequencing (NGS)	2	3	4	3,92	5	5
	Companion diagnostics	2	3	4	3,79	5	5
	Federated data infrastructures	2	3	4	3,82	4	5
Environmental factors	Climate change	1	1	1,5	2,03	3	5
	Pollution (air, water) and housing	1	1	2	2,11	3	5
	Occupational hazards	1	1	2	2,16	3	5
	Geographical location of citizens (infrastructural differences between regions)	1	2	3	3,00	4	5
	Concerns about the negative environmental impact of digital solutions	1	1	2	2,37	3	5
Political and policy-related factors	Democracy, the rule of law and fundamental rights that shape dignity, freedoms, equality and solidarity, citizens’ rights and justice	2	3	3	3,45	4	5
	Synergies from EU Policies against cancer (e.g. Europe’s Beating Cancer Plan or EU Council recommendations) and similar projects around the world	2	4	4	4,21	5	5
	National and international collaborations and partnerships	2	3,25	4	4,11	5	5
	Data protection laws (e.g. legal issues related to achieving broad patient consent and data sharing)	2	3	4	4,05	5	5
	National or EU policies that foster a secure environment for (collection, curation, analysis, storing…) sharing genomics data with relevant organizations, including cross-border	2	3	4	3,95	5	5
	Policies to protect the public from stigmatzsation and discrimination	1	3	3	3,18	4	5
	Translational research agenda in countries, e.g. through Comprehensive Cancer Centres in countries, and implementation of clinical guidelines	2	3	4	3,84	4,75	5
	Accountability and liability issues concerning the clinical decision support tools using AI	2	3	4	3,61	4	5

## Discussion

As this study has shown, to enable the fair and equitable implementation of precision medicine in Belgium in the future, it will be crucial to consider a range of contextual factors. First of all, survey respondents agree that the increasing incidence and prevalence of cancer cases in Belgium calls for precision medicine, as it offers personalized and targeted approaches to address the growing burden of the disease. Moreover, the survey results show that the availability of designated budgets for both reimbursement of precision medicine and research into its cost-effectiveness has been seen as crucial. Hence, in terms of economic factors, the availability of an allocated budget for reimbursement of personalized healthcare is vital to ensure that individuals can access and afford the benefits of precision medicine. Similarly, the presence of allocated funds for conducting studies on the cost-effectiveness of personalized medicine and translational research supports evidence-based decision-making, validating the value and impact of these approaches in routine cancer care.

Besides economic considerations, technological factors have been found important by the survey respondents. The use of IT solutions that facilitate secure and standardized transfer of relevant data to a unified technical platform is deemed crucial for seamless integration and interoperability of information, enabling comprehensive and coordinated care. Ensuring the quality of data used in precision medicine is also imperative for accurate decision-making and reliable outcomes. In this context, robust data governance and quality control mechanisms should be in place to support the implementation of precision medicine initiatives effectively. Semantic interoperability and data integration and harmonization are also key considerations to enable effective collaboration and knowledge exchange among healthcare providers, researchers, and stakeholders involved in precision medicine. Lastly, standardization of protocols, tests and nomenclature across hospitals and laboratories is found to be essential for consistent and reliable data interpretation in precision oncology, facilitating meaningful comparisons and enabling efficient decision-making.

Looking ahead, the increasing incidence and prevalence of cancer cases are expected to strongly contribute to the necessity for adopting precision medicine. This indicates the likelihood that precision medicine could be prioritized to address the growing demand for improved cancer care in the future. Second, the allocation of a budget specifically for the reimbursement of personalized healthcare is anticipated to enhance the chances of fair and equitable implementation. The recognition of the importance of funding in supporting accessible and affordable precision medicine solutions suggests a possibility of financial support being provided. Furthermore, the projected rise in healthcare expenditure within health systems may indicate increased investments in precision medicine. Additionally, the development of advanced IT solutions facilitating the secure transfer of relevant data to a uniform technical platform is expected to enhance the likelihood of fair and equitable implementation. This could enable seamless data integration and accessibility, promoting equitable access to personalized cancer care. Finally, the emphasis on reaching high-quality data in the health system is likely to enhance precision medicine implementation. Recognizing the anticipated increase in cancer burden in the coming years, below we offer recommendations on financial aspects, advanced IT solutions, and necessary technology for high-quality data, all aimed at making precision medicine accessible in routine cancer care in Belgium.

First, the allocation of a dedicated budget for reimbursing personalized healthcare will be crucial. Health systems are generally not designed to broadly adopt and scale such innovative medical solutions in routine care.^21^ To successfully integrate precision medicine into the Belgian health system, a concerted transformational effort across different stakeholder groups will be imperative. In this context, policymakers and decision-makers bear the responsibility to secure the necessary funding, while healthcare professionals should advocate for essential components such as biomarker testing, emphasizing why such solutions should be reimbursed by health systems.^22^ The rising expenditure in the Belgian health system underscores the importance of appropriately allocating resources to ensure equitable access to precision medicine while offering benefits for all individuals. Hence, policymakers in Belgium should prioritize the provision of financial support and incentives to ensure that individuals from all socio-economic backgrounds can access and afford the benefits of precision medicine. This can be achieved, for instance, through targeted funding schemes that specifically address the reimbursement of personalized healthcare, collaborations with pharmaceutical companies to negotiate affordable pricing for precision medicine treatments, and the establishment of reimbursement frameworks that consider the cost-effectiveness and long-term benefits of precision medicine interventions. By providing adequate economic support, barriers to access can be minimized, enabling fair and equitable distribution of precision medicine across the population.

In this context, it will be helpful to undertake continuous monitoring of health outcomes and evaluate the budgetary implications of precision cancer medicine associated with these efforts.^23^ Policymakers, research institutes, and the National Institute for Health and Disability Insurance (NIHDI) in Belgium should prioritize research funding in this area, providing resources for clinical trials, real-world evidence generation, and health economic evaluations. A comprehensive study evaluating the precision medicine health technology assessment (HTA) reports of NIHDI between January 2014 and January 2019 made the following recommendations to this aim: (i) implementing the linked evidence approach when direct evidence of clinical utility is not present; (ii) incorporating a bias assessment tool; and (iii) further specifying guidelines for submission and assessment to decrease the variability of reported evidence between assessment reports.^24^ Our findings support these recommendations. Indeed, by building a robust yet responsive evidence base, NIHDI can make informed choices regarding the implementation of precision medicine in Belgium, ensuring that resources are allocated to interventions that have proven efficacy, safety, and cost-effectiveness. This approach could ensure that precision medicine benefits are distributed equitably and that healthcare resources are used efficiently in clinical practice without extensive delays.

In terms of technological advancements, IT solutions that facilitate secure data transfer and interoperability will be essential for the effective implementation of precision medicine in routine care in Belgium. The implementation of precision oncology can be facilitated by the widespread adoption of data standards such as HL7 FHIR and mCODE if adopted by all entities involved in generating, transmitting, and receiving health information.^25^ An effective health data ecosystem that could function between the sectors will be increasingly important given that precision medicine heavily relies on real-world evidence. Hence, stakeholders should continue investing in the implementation of data standards that enable seamless exchange and integration of data across different systems and platforms to address the challenge of semantic interoperability and data integration. Collaborative efforts between healthcare providers, researchers, and IT professionals will be necessary to establish unified technical platforms that promote interoperability and enable comprehensive and coordinated care. Robust data governance policies and practices should also be put in place to ensure data security, privacy, and ethical use. By harnessing technological advancements and promoting interoperability, the seamless integration of data can be achieved, leading to more effective and equitable precision medicine implementation in Belgium.

Lastly, ensuring the quality of data used in precision medicine will be fundamental for accurate decision-making and reliable outcomes. Efforts in Belgium should be focused on establishing standardized imaging protocols, tests and nomenclature across hospitals and laboratories. This standardization enables consistent and reliable interpretation of data, facilitating meaningful comparisons and efficient decision-making. Indeed, the Belgian Society of Medical Oncology (BSMO) recognized years ago a specific challenge in personalized cancer treatment programs in Belgium: Laboratories working independently could achieve much better results if they collaborated in a larger, future-focused initiative. This would standardize testing methods and, ultimately, make treatment choices more consistent.^26^ Hence, harmonization initiatives should be encouraged, bringing together different stakeholders, including regulatory bodies, professional associations, and healthcare providers, to develop consensus guidelines and protocols. By implementing standardized practices and protocols, the quality and reliability of data used in precision medicine can be enhanced, ultimately leading to more equitable and effective patient care. To reach this aim, Belgium is increasingly joining forces with other European countries and collaborating in international initiatives.^27^^,^^28^

This study contributed to understanding the current landscape of precision medicine in routine cancer care in Belgium to proactively prepare for future challenges in this field. The methodology employed in this paper has several notable strengths. First, the adoption of a foresight approach provides a systematic and participatory mechanism for exploring future trends and their implications in healthcare. Second, the incorporation of the DESTEP framework, as guided by the European project PHIRI, lends the study a structured and comprehensive means for identifying contextual factors. Third, the use of a multidisciplinary group for validation and country experts for evaluation adds layers of rigor and credibility to the study. The extensive literature search strategy, including both peer-reviewed and gray literature, further strengthens the validity of the findings. However, our methodology also has a few limitations. The screening of peer-reviewed publications was restricted to the PubMed/MEDLINE database, which may limit the comprehensiveness of the literature search. Another constraint lies in the focus on Belgium, which may impede the generalizability of the findings to other health systems. Additionally, while the survey of experts enriches the study, the reliance on a specific group of experts could introduce some level of bias or narrow the perspectives represented. Nonetheless, our results may provide opportunities for further research and policy actions, ultimately contributing to ongoing advancements in precision medicine in Belgium and beyond.

## Conclusion

By addressing a complex and timely issue through a systematic lens, this work makes a meaningful contribution to the growing body of knowledge on precision medicine and public health foresight. While it establishes a comprehensive framework for current and future challenges, it also lays the groundwork for further research in Belgium and beyond. Future studies could focus on a comparative analysis with other European health systems to identify best practices and pitfalls in the implementation of precision medicine in a fair and equitable way. Moreover, ongoing evaluation of HTA methods in European countries and their alignment with precision medicine reimbursement mechanisms will be critical. Lastly, a deeper exploration of ethical considerations related to data privacy and equity will be crucial to ensure fair and responsible integration of precision medicine into health systems.

## Data Availability

The data underlying this article cannot be shared publicly due to the privacy of individuals that participated in the study. The data will be shared on reasonable request to the corresponding author. *Conflict of interest:* None declared. Key points The increasing incidence and prevalence of cancer in Belgium underscore the importance of adopting precision medicine as a personalized approach. Our study scrutinized influential contextual factors affecting the implementation of precision oncology in the Belgian health system. It contributed to understanding the key contextual factors expected to impact an equitable implementation of precision oncology in Belgium and provided targeted recommendations.
